# Using bodily postures to reduce anxiety and improve interoception: A comparison between powerful and neutral poses

**DOI:** 10.1371/journal.pone.0242578

**Published:** 2020-12-09

**Authors:** Felicitas Weineck, Dana Schultchen, Gernot Hauke, Matthias Messner, Olga Pollatos

**Affiliations:** 1 Department of Clinical and Health Psychology, Ulm University, Ulm, Germany; 2 Embodiment Resources Academy (ERA), Munich, Germany; Swinburne University of Technology Faculty of Health Arts and Design, AUSTRALIA

## Abstract

**Background:**

Previous research has shown that anxiety syndromes are highly prevalent among university students. Effective treatments are needed to reduce the burden of anxiety in this population. Powerful postures have been found to impact affective states, as well as interoception (i.e. the ability to perceive inner bodily signals). However, no previous study has compared the effects of powerful- and neutral postures in regards to anxiety and interoceptive ability.

**Methods:**

The first part of the study measured the single-session effect of adopting powerful- vs. neutral postures on students' (n = 57) interoceptive ability and *state* anxiety. The second part of the study measured the effect of adopting powerful or neutral postures twice daily for two weeks, on individuals' interoceptive ability and *trait* anxiety.

**Results:**

State anxiety decreased in both conditions whereas interoceptive accuracy only increased in the power posing condition after a single session. Interoceptive accuracy increased in both groups after two weeks of training.

**Limitations:**

The study included no comparison to a condition where individuals adopted their natural (i.e. usual) bodily posture.

**Conclusions:**

Embodiment interventions that include elements of adopting an open or expansive bodily posture whilst maintaining a self-focus, can help to reduce state anxiety and improve interoceptive accuracy in student populations. Power posing does not seem to be superior to holding a neutral posture to improve interoceptive accuracy or anxiety. One reason therefore could be that both conditions include the manipulation of self-focus and a postural change that diverges from individuals' normal posture.

## Introduction

Regarding university students, there is now a large body of evidence documenting the high burden of anxiety disorders and syndromes [1–3], with some studies showing a state anxiety prevalence of up to 81.7% and a trait anxiety prevalence of up to 85.6% [4]. Preliminary findings from a longitudinal study also highlight that self-reported anxiety disorders among students have doubled since 2008 [5]. Moreover, anxiety has been shown to correlate with lower perceived performance [6], lower self-esteem [7], procrastination [8], higher levels of stress and depression [9], lower psychosocial functioning and quality of life [10], as well as sleep disturbances [11]. Therefore, effective and efficient interventions are urgently needed to reduce anxiety symptoms and to improve the mental health in student populations [12, 13].

In this context, the use of *embodiment interventions* may be helpful. The embodiment approach to emotion generation proclaims that affective states can occur and be modified as a result of alterations in bodily postures, facial expression or the individual's voice [14–16]. Evidence for this assumption comes from studies highlighting the close connection between emotions and bodily movements [17–19]. Regarding anxiety, research by Lipnicki and Byrne [16] found that participants only experienced anticipatory anxiety when standing up, although not when lying down, before completing a demanding mental arithmetic task. In another study, Nair et al. [20] analysed word use during a stressful speech task. Individuals in an upright posture reported fewer words relating to fear than individuals in a slumped posture. Furthermore, research by Peper et al. [21] showed that individuals who adopted an upright bodily position, took a breath, and then reframed their negative thoughts were significantly more successful in reducing their anxiety compared to individuals who reframed their negative thoughts without adopting an upright bodily posture. However, the evidence regarding anxiety and posture remains mixed. Others studies that have investigated the influence of upright bodily posture compared to reclined bodily posture on self-reported anxiety found no beneficial effects [22–24]. One reason therefore could be that the design of the experiments and the chosen outcome variables varied greatly between the studies. For example, they focused either on a specific type of anxiety, such as social anxiety [24], anticipatory anxiety [16] or anxiety in general [21], or measured anxiety whilst the posture was adopted [25] or after a performance task in which the posture was no longer held [24]. Also, the manipulations of the postures differed in regards to their focus on the whole body [16, 22], the upper body [20, 21, 26] or only parts of the body [25].

To our knowledge, no previous study specifically investigated the effect of *power posing* [27], on individuals' state- and trait anxiety using the validated State-Trait Anxiety Inventory [28]. Power posing refers to the adoption of an expansive and dominant bodily posture [27]. From a theoretical point of view, power posing should decrease state anxiety in the short-term, as power posing has been shown to increase state self-esteem [29], which in turn, has been found to be negatively correlated with anxiety [30]. Also, straight postures (as opposed to slumped postures) seem to foster mood recovery [14, 25]. Furthermore, expansive and open bodily postures have been associated with emotions such as pride [31], whereas slumped postures have been found to correlate with emotions such as guilt [32, 33]. Designing effective embodiment interventions that can easily be applied in everyday life might offer students novel coping strategies for reducing their anxiety levels.

Another aspect highly relevant in the context of emotion regulation is *interoceptive ability* [34]. Interoception refers to the perception and processing of internal bodily signals [35]. Previous research could show that interoceptive accuracy (IAcc) facilitated the down-regulation of affect [36]. For example, individuals with higher IAcc made more use of reappraisal and suppression strategies, when regulating their emotions, compared to individuals with lower IAcc [37]. From a theoretical point of view, the induction of power via a bodily posture should increase interoceptive ability, as power has been linked to increases in *self-focus*. From an evolutionary perspective, powerless individuals are more likely to shift their attention outwards to detect potential threat or to gain access to resources [38]. Therefore, they have less capacity to divert their attention inwards [39]. Powerful individuals, on the other hand, have a higher capacity to shift their attention inwards, as they are less dependent upon others for personal resources and are therefore less likely to divert their focus outwards towards less dominant individuals [40]. As a consequence, if someone is more focussed on him or herself, the idea is that he or she is more likely to notice bodily signals [41]. In keeping with this assumption, previous research has shown that individuals who are primed with power find it more difficult to engage in other-oriented perspectives than powerless individuals [42], are less distracted by external information [43] and report trusting their 'gut feeling' when it comes to decision making [43, 44]. Regarding IAcc in particular, Kunstman et al [45] found that power priming through words or writing up powerful memories increased IAcc in those with high levels of body dysmorphic symptomatology. Furthermore, Moeini-Jazani et al. [46] showed that IAcc could also be increased through the induction of social power via a powerful role play (manager vs. manager-subordinate). In a previous pilot study, we found that IAcc could be improved through a single power posing session [47]. This body-focused approach appears to be particularly promising as powerful postures have been shown to have stronger effects on the activation of implicit power than powerful roles [48]. However, our study did not reveal whether the effect of increased IAcc could be upheld when comparing power posing to neutral posing and we did not investigate the effects of power posing on anxiety. Therefore, the current study will expand previous research by comparing neutral- and power posing in regards to interoceptive abilities (i.e. IAcc, interoceptive sensibility and interoceptive awareness) and anxiety. This is also important, as research comparing powerful to neutral postures remains scarce [49, 50]. The majority of previous studies have used contractive postures as a comparison group [51]. This is problematic, as it makes it difficult to identify the true positive effect of powerful postures and to disentangle it from the negative effect of contractive poses [49].

To address the research questions, participants underwent a single power posing- or a single neutral posing session, as well as two weeks of daily power posing- or neutral posing practice. Primary outcomes were measures of anxiety and interoceptive ability. We hypothesised that (I) One session of power posing would increase individuals' interoceptive abilities and decrease their state anxiety scores compared to neutral posing; (II) Two weeks of daily power posing would increase individuals' interoceptive abilities and decrease their trait anxiety scores compared to neutral posing.

## Methods

### Participants

Sixty-four healthy students were recruited at Ulm University. In return for their participation, they received course credit. Exclusion criteria were a present psychiatric or physical disorder, age under 18 and specific experience or knowledge regarding power posing. Seven participants were excluded from the study due to the diagnosis of chronic physical illness (n = 1), knowledge about power posing (n = 1) or because they did not adhere to the training protocol (n = 5). This exclusion left a total of 57 (12 male) participants. Participants had a mean age of 22.70 (*SD* = 3.88) and a mean BMI of 21.86 (*SD* = 2.72). None of the participants were taking medication, except contraceptives (n = 8). The number of participants in our study was in keeping with the a priori power analysis. Based on data from a similar study by Moeini-Jazani [46] (n = 135), comparing powerful, neutral and powerless conditions, their effect size (ES) was considered to be medium using Cohen's [52] criteria. With an alpha = .05 and power = 0.80, the projected sample size required for this effect size (G*Power 3.1.9) was n = 46 for the simplest within-group comparison.

### Questionnaires

After having signed up for the study, participants received an online questionnaire via email, collecting health-related and demographic information (including age, BMI, educational background, level of fitness, hours of sport per week, smoking and previous experience with body-centred interventions). Also, the German version of the Patient Health Questionnaire (PHQ-D) [53] was administered.

Furthermore, the German version of the State-Trait Anxiety Inventory (STAI) was applied [54]. It is a validated measure, containing a total of 40 items, assessing different dimensions of anxiety on a 4-point scale, ranging from 'almost never' to 'almost always'. The distinction is made between state and trait anxiety. State anxiety indicates how the participant currently feels, i.e. it reflects a fluctuating emotional state that can vary depending on the situational context. Trait anxiety, on the other hand, reflects the individuals' proneness to anxiety in general. The subscales for the dimensions include 20 items each and scores can range from 20–80 respectively (with higher scores reflecting higher anxiety).

After two weeks of training the power- or neutral posing, participants were also given an evaluative questionnaire constructed by the authors to assess training compliance. Individuals were asked how many times they practised daily and could indicate their answers on a 4-point scale ('less than once per day'; 'once per day'; 'two times per day'; 'more than two times per day'). Please note that this study was part of a larger research project. Therefore, several other questionnaires were administered (e.g. the Multi Motive Grid by Sokolowski et al. [55] and the Personal Sense of Power Scale by Anderson et al. [56]), which will not be addressed here, as they will be published in another article with a focus on a clinical sample.

### Measurements of interoceptive ability

#### Interoceptive Accuracy (IAcc)

IAcc reflects performance on behavioural tests of interoception [57]. The heartbeat tracking task (HBTT) [58] was used to assess individuals' IAcc. It is one of the most widely used measures of interoception [59, 60], is easy to administer and has good test-retest reliability [61–63]. It also correlates with other measures of heartbeat detection [64, 65] and neural markers of interoception, such as heart-beat evoked potential [66]. The task included four heartbeat-counting trials, which were presented in a fixed order. Participants were instructed to silently count their heartbeats during these intervals (35s, 45s, 25s and 60s). In between each interval was a 45s break, where they were asked to report the number of counted heartbeats. The experimenter gave a start and stop cue at the beginning and the end of each counting interval. Before the task began, participants were instructed not to take their pulse or to use any other form of manipulation to support the counting of their heartbeats. Also, no prior information regarding the length of the counting phase was given, and participants received no feedback on the quality of their performance. Heartbeat signal was recorded with the BioPac Model MP150, using the software AcqKnowledge 4.0 (Biopac Systems, Inc., Goleta, CA). For the recording, three electrodes were placed on the right clavicle, the lower left part of the ribcage and the right hip. IAcc was calculated as the mean heartbeat perception score according to the following transformation:

$$\frac{1}{4}\sum (1-\frac{\left(\left|recorded\;heartbeats-counted\;heartbeats\right|\right)}{recorded\;heartbeats})$$

IAcc scores could range from 0 to 1. Higher scores indicate smaller differences between the counted and recorded heartbeat and thus better IAcc.

#### Interoceptive Sensibility (IS)

IS reflects the individuals' self-confidence relative to his or her objective performance on the HBTT [57]. After each interval of the HBTT, participants were asked to rate their confidence in their performance on a scale ranging from one to ten (1 = not confident at all; 10 = fully confident). As a secondary measure of IS, the Body Perception Questionnaire [67] was applied. This measure has previously been used to assess IS [57, 68]. The BPQ awareness subscale includes 45 items reflecting different bodily sensations. Examples of the items are 'I am aware of how fast I am breathing' or 'I am aware of muscle tension in my arms and legs'. Participants can indicate their awareness of each sensation on a 5-point scale ranging from 'never' to 'always'.

#### Interoceptive Awareness (IAw)

IAw refers to the concordance between IAcc and IS measures [57]. To operationalize IAw, we applied the 'percent of maximum possible' or POMP scoring method [69]. This way of expressing IAw has been applied in a previous article by Weineck et al. [47]. A POMP score is the result of a linear transformation of any raw metric into a 0 to 100 scale. In this study, IAcc and confidence scores (= IS) were converted into POMP scores to make them comparable. Thereby the following formula was used: POMP = [(observed—minimum) / (maximum—minimum)] x 100. 'Observed' represented the observed score for a single case, 'minimum' reflected the minimum possible score on the scale, and 'maximum' reflected the maximum possible score on the scale. After the conversion, the absolute difference between interoceptive accuracy- and sensibility POMP scores was calculated. At this stage, higher scores reflected higher discrepancies between interoceptive accuracy and confidence and thus, lower interoceptive awareness. For the scaling of the awareness score to be intuitive, i.e. for higher scores to represent higher awareness, the calculated absolute difference between POMP scores was again subtracted from 100. This transformation results in an awareness score between 0 and 100. Representing IAw in the form of POMP scores has the benefit that an individual awareness score, reflecting the correspondence between IAcc and confidence, can be generated for each participant and, therefore, changes over time can easily be recorded.

Furthermore, the Pearson correlation r, between IAcc and confidence was used as an index of interoceptive awareness [70].

### Embodiment interventions—bodily postures

**Fig 1:**
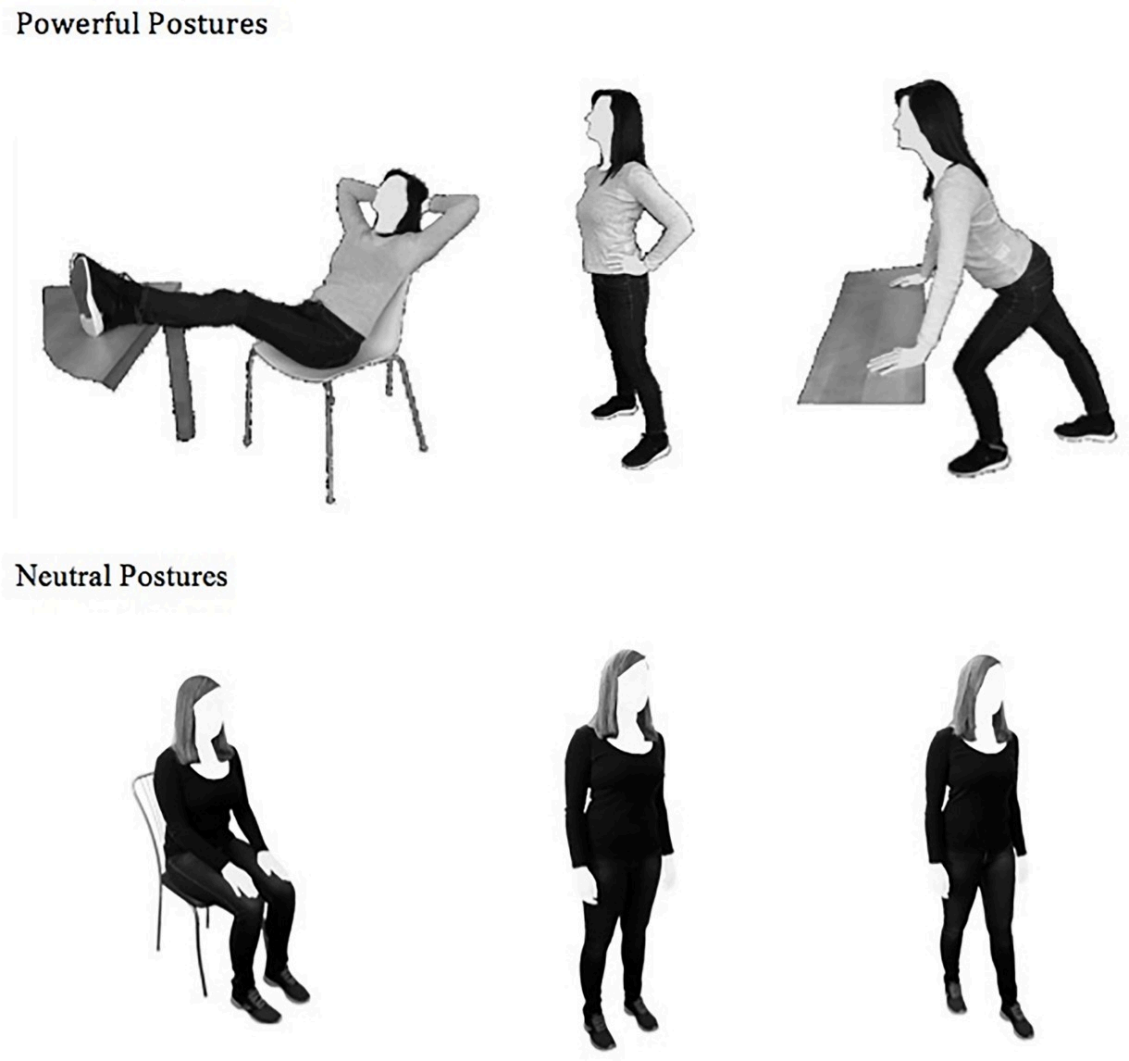
Display of powerful and neutral postures in the order of their application.

Refer to Fig 1 for a display of the powerful and neutral postures. All postures are presented in the order of their application.

#### Powerful postures

The three different powerful postures were selected from studies on bodily displays of power [27, 71, 72]. They were the same ones as those used in our previous pilot study on the effects of power-posing on interoceptive abilities [47]. Each posture contained several elements associated with power such as expansive positions of the arms and legs, the chin and gaze that were tilting upwards and the adaptation of an open bodily position [73]. The order in which the postures were adopted was fixed, and each pose was held for 45 seconds. This duration was chosen, based on previous studies showing beneficial effects after 60 to 120 seconds of power posing [51]. An audiotape (female voice), that lasted for approximately six minutes, guided individuals through each posture. To ensure that the participants raised their chin correctly whilst practising the poses, they were asked to look at a cross on the wall, placed one hand length above their individual height, at a 1.5-meter distance. Participants were also instructed to continually shift their attention between three anchor points (feet; sternum; chin) to ensure that they did not automatically fall back into their initial or a slumped posture.

#### Neutral postures

The three different neutral postures were closely linked to the powerful postures in terms of bodily alignment (i.e. if the person in the powerful posture was sitting down, the person in the neutral posture was also sitting down), however, they lacked the expression of bodily power [74]. As an example, in posture number two (see Fig 1), the participant had to stand up, however, instead of expanding the arms sideward, the arms were left hanging parallel to the upper body. Also, instead of tilting the head upwards, the participants were asked to look directly ahead of them. The fixation cross on the wall was placed on their individual eye level at a 1.5-meter distance. Similar to the powerful postures, the order in which the neutral postures were adopted was fixed, and each pose was held for 45 seconds. Here as well, an audiotape (female voice) was played, that lasted for the same amount of time as for the powerful postures, guiding individuals through each posture. Participants were also asked to continually shift their attention between three anchor points (feet; sternum; chin).

### Procedure

The study was conducted in accordance with the Declaration of Helsinki, and ethical approval was obtained from the Institutional Review Board (IRB) of Ulm University. Before testing, informed consent was collected from all participants according to IRB requirements. Data collection took place at the laboratories of the Clinical and Health Psychology Department of Ulm University. Participants were informed that the study aimed to investigate the effects of cervical spine training on different aspects of health. This cover story was used to avoid potential power priming and response bias.

Before the testing in the laboratory, participants filled out an online questionnaire collecting demographic and health-related data. After the testing of individuals' interoceptive and anxiety scores at baseline in the laboratory, all participants were randomly assigned to two groups via blocked randomization. The power posing group (n = 29) practised power posing for one session, whilst the neutral posing group (n = 28) adopted neutral postures for one session. Participants were tested one-by-one. The poses were shown on a picture and subsequently presented in person by the experimenter. Individuals were then asked to adopt the poses themselves. After a short training session, that endured for as long as each participant needed to understand and apply the postures correctly, the audiotape was played, guiding the participants through the movements. The heartbeat tracking task (HBTT) [58], a question on individuals' confidence on the task, as well as a fixed-format questionnaire, were administered before (baseline) and after each session (T1). The second part of the study measured the effects of training either the powerful- or the neutral postures twice a day for two weeks at home. The power posing group (n = 29) underwent power posing practice twice daily for two weeks, whilst the neutral posing group (n = 28) underwent neutral posing practice twice daily for two weeks. Participants were instructed to adopt the postures once in the morning and once in the evening. To increase compliance with the training routine participants were given diary charts. Participants’ interoceptive ability was tested again after one week of training (T2), as well as two weeks of practice (T3).

#### Data analysis

All data were analyzed using IBM SPSS Statistics 25 software (SPSS, Chicago, IL). Mixed ANOVAs were calculated to investigate the effects of embodiment interventions (2 levels: power posing vs. neutral posing) on interoceptive ability and anxiety over time (2 levels: either baseline vs. after a single training session, or 4 levels: baseline, T1, T2, T3). Mauchly's test of sphericity was performed for each analysis and the results were adjusted accordingly if the test was significant. P-value significance level cut-off was adjusted for multiple comparisons using the Bonferroni correction method. Missing data varied slightly between measures, and the numbers of cases are reported for each analysis. The effect size for the tests with mixed ANOVA was expressed in terms of partial eta squared (η^2^part) and divided into the categories small effect size (η^2^part .01 to < .06), medium effect size (η^2^part .06 to < .14), and large effect size (η^2^part > = .14) [52].

## Results

### Demographics and questionnaire data

For demographic information and relevant health-related outcomes refer to Table 1.

**Table 1:** Demographic information at baseline.

	Baseline												
Variable	Neutral Posing					Power Posing					Test		
	*M*	(SD)	*[n]*	*%*	*n*	*M*	(SD)	*[n]*	*%*	*n*	*T/X^2^*	*df*	*p*
Age	22	(2.07)	[28]			23.4	(5.00)	[29]			-1.370	38	.179
BMI (kg/m^2^)	22	(2.65)	[28]			21.7	(2.82)	[29]			0.369	55	.713
Level of Fitness	58.7	(17.41)	[28]			56.5	(17.42)	[29]			0.482	55	.631
Hours of sport per week	3.5	(1.91)	[28]			2.4	(2.40)	[29]			1.817	54	.075
Females				75	21				82.8	24	0.516	1	.473
Individuals who smoke				28.6	8				24.1	7	0.144	1	.704
Current significant burden in life				32.1	9				41.4	12	0.552	1	.470
Experience with body interventions				42.9	12				34.5	10	0.442	1	.516

**Note.** *M = Mean; SD = Standard Deviation; n = Number of Participants*.

Score Ranges. Level of Fitness (0–100)

### Effects of power- or neutral posing on state and trait anxiety

State and trait anxiety mean scores sorted by condition and time points are visualized in Table 2. Regarding the single session effect of power posing or neutral posing on state anxiety, a mixed ANOVA was calculated with the factors condition (neutral posing, power posing) and time (baseline; after one session). Results showed a significant effect of time on *state* anxiety, *F*(1, 55) = 9.07, *p* = .004, with a large effect (part.η^2^ = .142). No significant effect of condition (*F*(1,55) = 0.42; *p* = .520), and no significant interaction effect between time and condition were found, *F*(1, 55) = 0.13, *p* = .723. One-sided post hoc tests with Bonferroni correction revealed a significant decrease in state anxiety from baseline (*M* = 36.10; *SD* = 7.76) to T1 (*M* = 34.03; *SD* = 8.18) for power posing (t(28) = 2.119, *p* = .043), as well as for neutral posing (baseline: *M* = 37.21; *SD* = 8.26; T1: *M =* 35.58; *SD* = 8.16), *t*(27) = 2.219, *p* = .035.

**Table 2:** Means and standard deviations of anxiety and interoceptive measures.

		Measurement points			
		Baseline	T1	T2	T3
Variable	Group	*M* (SD) [n]	*M* (SD) [n]	*M* (SD) [n]	*M* (SD) [n]
Anxiety State	Neutral posing	37.21 (8.26) [28]	35.58 (8.16) [28]	-	-
	Power posing	36.10 (7.76) [29]	34.03 (8.18) [29]	-	-
	Total	36.65 (7.96) [57]	34.80 (8.13) [57]	-	-
Anxiety Trait	Neutral posing	39.08 (8.62) [26]	-	37.58 (8.44) [26]	38.27 (8.87) [26]
	Power posing	40.93 (9.36) [28]	-	41.32 (9.60) [28]	41.04 (9.53) [28]
	Total	40.04 (8.98) [54]	-	39.52 (9.17) [54]	39.70 (9.24) [54]
Interoceptive Accuracy	Neutral posing	0.66 (0.16) [24]	0.69 (0.16) [24]	0.71 (0.19) [24]	0.73 (0.18) [24]
	Power posing	0.63 (0.19) [26]	0.68 (0.18) [26]	0.69 (0.17) [26]	0.71 (0.16) [26]
	Total	0.64 (0.17) [50]	0.68 (0.17) [50]	0.70 (0.17) [50]	0.72 (0.17) [50]
Interoceptive Sensibility	Neutral posing	4.04 (1.66) [26]	4.17 (1.50) [26]	4.17 (1.76) [26]	4.26 (1.46) [26]
(Confidence scores)	Power posing	4.83 (1.47) [26]	5.14 (1.63) [26]	5.11 (1.51) [26]	5.32 (1.42) [26]
	Total	4.43 (1.60) [52]	4.66 (1.63) [52]	4.64 (1.69) [52]	4.79 (1.52) [52]
Interoceptive Sensibility (BPQ)	Neutral posing	2.74 (0.88) [26]	2.75 (0.89) [26]	2.84 (0.90) [26]	2.78 (0.91) [26]
	Power posing	2.54 (0.81) [28]	2.62 (0.91) [28]	2.67 (0.90) [28]	2.69 (0.99) [28]
	Total	2.63 (0.84) [54]	2.68 (0.89) [54]	2.75 (0.89) [54]	2.73 (0.94) [54]
Interoceptive Awareness	Neutral posing	68.45 (18.50) [24]	66.40 (19.13) [24]	63.08 (18.98) [24]	60.81 (16.26) [24]
	Power posing	78.00 (16.21) [26]	77.49 (16.13) [26]	74.26 (17.53) [26]	72.82 (15.19) [26]
	Total	73.42 (17.83) [50]	72.17 (18.33) [50]	68.89 (18.91) [50]	67.06 (16.69) [50]

**Note.** *M = Mean; SD = Standard Deviation; n = Number of Participants*.

Score Ranges. Body Perception Questionnaire (BPQ) (1–5); Interoceptive Accuracy (0–1);Interoceptive Sensibility (1–10); Interoceptive awareness (0–100)>

Anxiety State (STAI-S) (20–80); Anxiety Trait (STAI-T) (20–80).

Regarding the training effect of power posing and neutral posing on trait anxiety, a mixed ANOVA was calculated with the factors condition (neutral posing, power posing) and time (baseline; T2, T3). Results showed no significant effect of time on trait anxiety, *F*(2, 104) = 0.66, *p* = .520. No significant effect of condition (*F*(1, 52) = 1.33; *p* = .253), and no significant interaction effect between trait anxiety and condition was found, *F*(2, 104) = 1.88, *p* = .158.

### Effects power- or neutral posing on interoceptive ability

**Fig 2:**
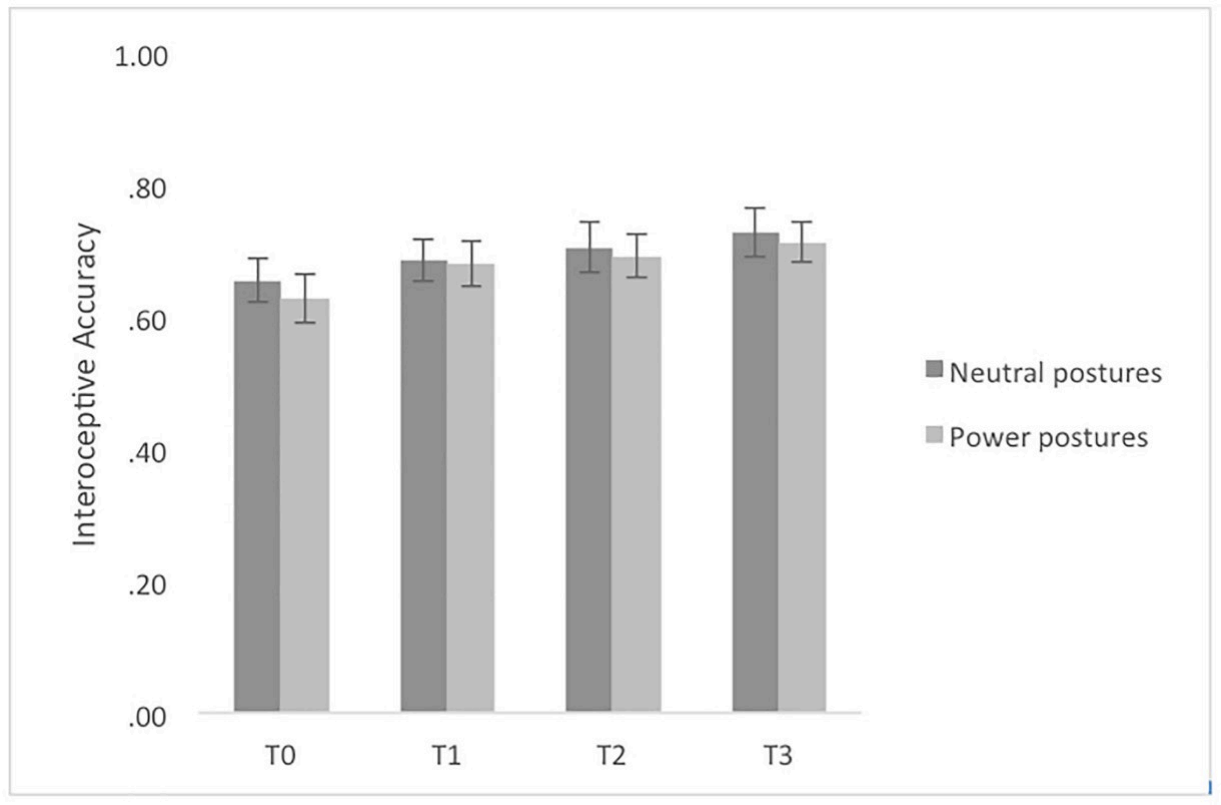
Interoceptive accuracy mean scores sorted by measurement point for powerful and neutral postures. Note. Error bars represent SE.

Regarding the training effect of power posing or neutral posing on interoceptive ability, mixed ANOVAs were calculated with the factors condition (neutral posing, power posing) and time (baseline; T1, T2, T3). Results showed a significant effect of time on IAcc, *F*(2.68, 128.78) = 6.62, *p* = .001, with a medium effect size (part.η^2^ = .121). No significant effect of condition (*F*(1,48) = .12; *p* = .734), and no significant interaction effect between time and condition were found, *F*(2.68, 128.78) = 0.13, *p* = .927. Post hoc tests with Bonferroni correction revealed that there was a significant increase in IAcc from baseline to T1 (*p* = .038), to T2 (*p* = .031) and T3 (*p* = .001) for posing in general (Table 2 and Fig 2). Regarding the individual conditions, one-sided post hoc tests with Bonferroni correction revealed that there was a significant effect of time on IAcc for power posing from baseline to T1 *t*(26) = -3.076, *p* = .010 and from baseline to T3 (*t*(26) = 3.768, *p* = .002). Regarding neutral posing, there was no significant effect of time from baseline to T1, *t*(25) = 1.948, *p* = .126, however, we found a significant effect of time from baseline to T3, *t*(23) = -2.512, *p* = .038.

**Fig 3:**
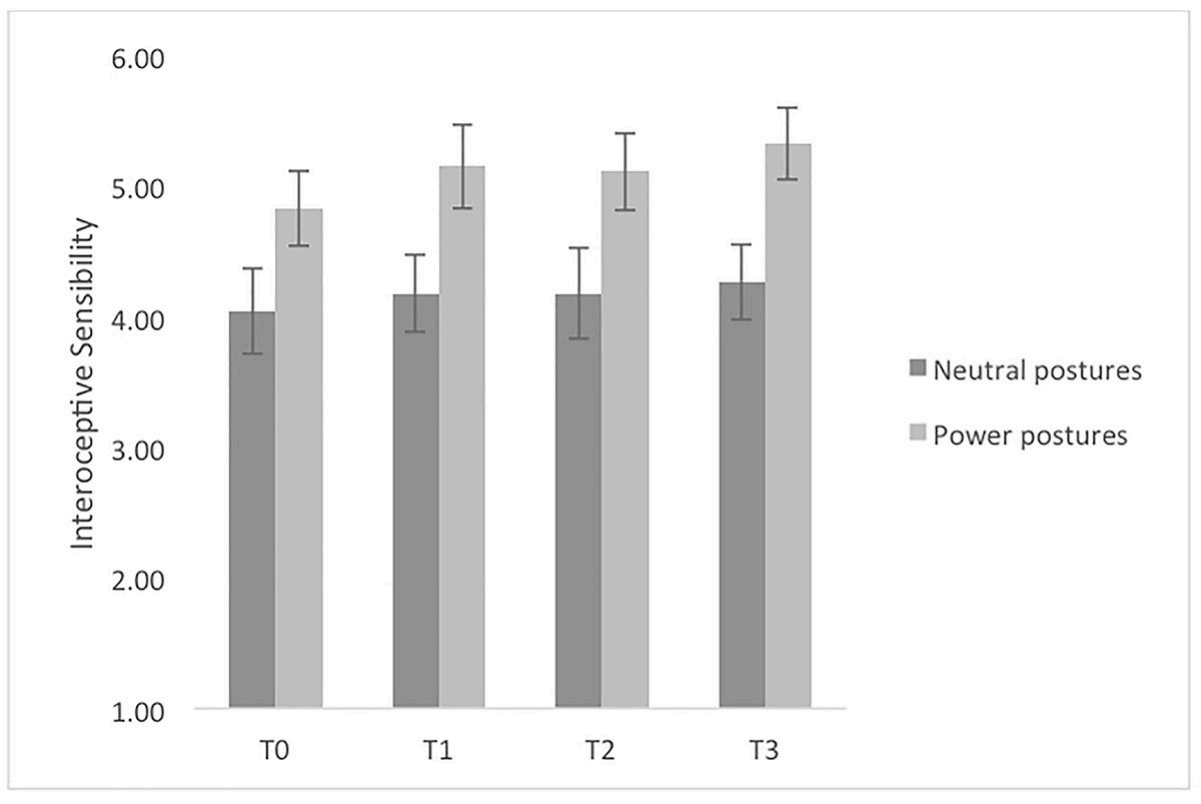
Interoceptive sensibility mean scores sorted by measurement point for powerful and neutral postures. Note. Error bars represent SE.

Regarding IS, measured by confidence rating, no significant effect of time, *F*(3, 150) = 2.03; *p* = .112, no significant effect of condition (*F*(1,50) = 5.72; *p* = .021), and no significant interaction effect between time and condition, *F*(3, 150) = .30; *p* = .829 was found (Fig 3). When looking at interceptive sensibility with the BPQ measure, also, no significant effect of time, *F*(1.51, 78.37) = 2.06; *p* = .146, no significant effect of condition (*F*(1,52) = .37; *p* = .544), and no significant interaction effect between time and condition (*F*(1.51, 78.37) = 0.37; *p* = .635) was found. No significant effect of time (*F*(2.71, 129.88) = 4.32; *p* = .008), no significant effect of condition (*F*(1,48) = 6.73; *p* = .013), and no interaction effect between time and condition (*F*(2.71, 129.88) = 0.13; *p* = .927), was observed regarding interoceptive awareness. The correlation between interoceptive accuracy and confidence was not significant at T3 (r = .118; *p* = .174).

## Discussion

The central aim of the study was to investigate whether power posing and neutral posing had an effect on individuals' anxiety levels and interoceptive ability. In particular, it was assessed whether power posing was superior to neutral posing in regards to these primary outcome measures. Fifty-seven healthy students were randomly assigned to two groups (power posing vs. neutral posing). Firstly, we measured the impact of a single power posing session and a single session of neutral posing on interoceptive ability and state anxiety. Then, we measured the effects of two weeks of daily training of the powerful or neutral postures on individuals' interoceptive ability and trait anxiety. The main finding was that there was a significant main effect of time on IAcc, thus, IAcc improved in the short-term and after two weeks through posing. We also found a significant effect of both embodiment interventions on state anxiety after a single session of adapting the powerful or neutral poses. No effects on the other dimensions of interoception were found.

Firstly, the hypothesis that power posing was superior to neutral posing regarding state anxiety was unsupported, as there was no significant main effect of condition. One explanation therefore could be that the neutral posing condition also entailed a treatment effect. The postures were neutral in the sense that they lacked the elements of perceived dominance. We did not, however, control for how individuals were usually standing or sitting. Thus, the poses the participants adopted in the neutral condition may have included elements of openness and expansiveness that were novel to the participants. Posture two (see Fig 1), for example, has been used in previous research as a neutral comparison posture [74]. However, when conducting a literature review, we also found that it is closely related to a foundational yoga posture called *tadasana*, which has been found to alter heart rate variability [75]. In this context, it is also noteworthy, that both, the powerful and the neutral postures, contained features that were opposed to those used to induce fear in previous research [19]. Thus, adopting an open, non-restrictive bodily posture may evoke anxiolytic responses, independent of how expansive it is. Furthermore, as the neutral poses were adopted one after the other, they may have represented a form of *meditative movement*, which is characterized by a focus of awareness on the body and some form of prescribed or spontaneous movement [76, 77]. Research on meditative movement has shown, that moving the body in space whilst shifting attention towards the self can help to ameliorate anxiety and depression [77]. Thus, in the future, it would be valuable to include usual postures (i.e. postures that do not require the individuals to maintain an instructed position) as well, to disentangle the effects of the applied embodiment interventions.

Although power posing was not superior to neutral posing, the study revealed that power posing and neutral posing reduced individuals' *state* anxiety in the short-term. Regarding the effect of power posing, this observation is in keeping with research highlighting that the induction of power seems to foster subjective feelings of power [78], which, in turn, have been found to be beneficial for the reduction of anxiety [79]. As an underlying mechanism, it could also be that the powerful postures activated the individuals' behavioural approach system, which has been linked to positive affect, as opposed to the behavioural inhibition system, which has been associated with avoidance-related affect (e.g., anxiety, fear) [38]. Anderson et al. [80], for example, found that participants with high feelings of power experienced more emotions such as pride, and fewer emotions such as fear, compared to participants with low feelings of power. They were also more likely to perceive rewards and less likely to perceive threats. Thus it can be speculated that the features of the power poses (e.g. leaning forward) may have induced a particular physiological activity that, in turn, influenced emotional processing [81]. In this context, Hackford et al. [82], found that individuals who walked in an upright posture showed significantly lower systolic blood pressure and galvanic skin response than those who walked in a slumped walking posture. In addition, the upright walking group showed improved psychological states including less low arousal negative affect, less sleepiness and less pain. To get a better understanding of the underlying physiological mechanisms that supported the observed effects in our study, it would be beneficial to include physiological measures in future research, for example by assessing individuals' skin conductance response whilst holding the postures [83].

Considering the results on anxiety further, no effects on trait anxiety for posing in general were found, when comparing the time points against each other. One explanation could be that two weeks of training is not long enough to achieve changes in trait anxiety through posing. Previous studies aiming at a reduction in trait anxiety have often designed interventions that took place for longer periods, e.g. ten weeks [84]. Also, it is important to note that data collection took place during the exam period, and students may have experienced greater test anxiety. As Spielberger [85] pointed out that test anxiety could be a form of situation-specific trait anxiety [86], higher levels of test anxiety may have confounded individuals' trait anxiety scores during the time of testing.

Regarding interoceptive accuracy, we found that power posing significantly increased individuals' IAcc in the short-term and after two weeks of training, when compared to baseline. This finding is in keeping with our previous study on embodied power [47], as well as research on power priming, highlighting that the induction of power appears to foster individuals' IAcc [45, 46]. In this context, it is noteworthy, that neutral posing did not significantly improve IAcc from baseline to T1. However, here as well, we did not find support for the hypothesis, that power posing is superior to neutral posing in regards to IAcc, as we did not find a significant main effect of the conditions.

Regarding our hypothesis that power posing would be superior to neutral posing in regards to IAcc after two weeks of training, this hypothesis was also unsupported, as improvements occurred in both groups and there was no significant main effect of the conditions. One reason could be that through the open bodily features that are included in both postures categories, a state of non-threat and relaxation is evoked, which fosters attention to bodily signals through the absence of stress-related arousal [82, 87, 88]. An alternative explanation could be that both conditions included the element of *self-focus*, which is probably induced whilst ensuring that the poses are adopted correctly (i.e. the focus on the feet, the sternum and the position of the chin). Previous experiments could show that self-focus increases interoceptive accuracy [46]. For example, Ainley and colleagues found that individuals' IAcc could be improved when individuals looked at themselves in a mirror [89], at a photograph of themselves, or processed self-relevant words [90]. Thus, in the future, it could be useful to compare the postures used in this study to two conditions, where individuals are not instructed to hold particular postures but to simply stand or sit in their usual posture, *with or without* shifting their attention towards the three anchor points. This could help to gain a deeper understanding of the influence of *self-focus* on the observed effects. The improvements in both groups after two weeks are noteworthy, as they highlight a possible training effect of IAcc. Regularly adopting open bodily postures and moving the body in space (i.e. embodiment interventions) seem to benefit IAcc, as the mean values increased steadily over time in both conditions. This is an important finding regarding the current debate whether and how interoceptive ability can be improved through the regular practice of a particular intervention [91, 92]. In this context, Fischer et al. [93] found a significant positive effect of a body scan intervention on IAcc after eight weeks of practice. Similarly, Bornemann et al. [63] reported improvements in IAcc after six to nine months of contemplative practice. However, other research found no training effect of IAcc through mindfulness after two months [94]. To get a better understanding of the training effects of power posing or neutral posing it would be interesting to investigate, whether IAcc would continue to improve over prolonged periods of practice (e.g. eight weeks).

Looking at the current evidence-base for powerful postures in general, there have been mixed results regarding their effectiveness. Supporting evidence seems to depend on the chosen outcome variables. For example, a recent p-curve analysis, including 55 studies, by Cuddy et al. [78] found no support for so-called non-EASE variables (including e.g. hormones, pain threshold or performance in a job interview). However, they found supporting evidence for EASE variables (including emotions, affect and self-evaluations). Therefore, our findings regarding the effects of powerful postures are in keeping with this recent analysis as they highlight benefits on affective states as well as the perception of bodily signals, which are an important prerequisite for emotion processing [36].

The study has several limitations. Firstly, we did not collect any qualitative or quantitative data on the subjective experience of the postures. It could be that participants preferred one posture to the other and one group may have enjoyed practising the poses more than the other. Regarding this point, it would be beneficial to also include likability ratings in future research to understand which intervention receives higher preference ratings. Also, comparing powerful postures to individuals' usual or natural postures, as aforementioned, might help to get a better understanding of the underlying mechanisms of the observed effects. Furthermore, there may have been demand characteristics that could have impacted the results. However, as we tried to minimize these by using a cover story, removing all headlines from the questionnaires and embedding the anxiety questionnaires in a series of other questionnaires to avoid priming the individuals with the main objective of the study. Nevertheless, demand characteristics could have played an important role, especially as recent studies have highlighted the influence of the social context on bodily posture effects (see [51, 95, 96]). Thus, the results of this study should be interpreted baring this possible confounding variable in mind. For future research, it would also be valuable to adopt the different postures once with and once without the experimenter present to get a better understanding of how experimenter presence or absence may have impacted the results. Considering limitations further, the heartbeat detection task has been criticised for being susceptible to non-interoceptive influences, such as knowing or guessing ones' heart rate, time estimations or practice effects [97, 98] and we did not control for these possible confounding factors. In this context, it is noteworthy that several recent studies that included a time estimation task, did not find a positive relationship between time estimates and interoceptive ability, and controlling for time estimates did not change the relationship between IAcc and other outcomes [99, 100]. It is therefore unlikely that time estimates confounded the results. Also, regarding practice effects, the post hoc tests revealed no significant improvement in the neutral power posing group in the short term. If practice effects had impacted the results, this should have resulted in an increase in IAcc in the neutral posing group at T1. Also, other studies that applied the HBTT at different time points either did not find significant improvements over time [94, 101] or did find variations in IAcc depending on the manipulation used [89, 93]. These observations speak against the influence of practice effects. Finally, the study was conducted using a sample of university students and the results may not be generalizable to individuals with a diagnosed anxiety disorder. However, previous studies have chosen a cut-off score of >40 on the STAI for minor and major anxiety symptomatology [102] and it is noteworthy that our student sample displayed a baseline mean score of 40.04. Thus, based on this observation the results of this study may be generalizable to individuals with a similarly high trait anxiety score.

Regarding clinical implications, the study highlights that, although power posing is not superior to neutral posing, adopting open, mindful postures and moving the body in space seem to be beneficial for interoceptive accuracy as well as state anxiety. This observation is particularly relevant to consider when designing embodiment interventions aiming at the reduction of anxiety in students.
